# Design and simulation of the potential of lead-free Ag_3_Bi_1.1_I_6.3_ perovskite solar cells with different charge transport for energy enhancement

**DOI:** 10.1039/d5ra04146e

**Published:** 2025-08-04

**Authors:** Md Aminul Islam, M. Khalid Hossain, M. Shihab Uddin, Apon Kumar Datta, Sahjahan Islam, Pardeep Singh Bains, Rohit Sharma, A. Rajiv, Abdullah M. S. Alhuthali, Magda H. Abdellattif, D. K. Dwivedi, Rajesh Haldhar

**Affiliations:** a School of Electrical, Computer and Energy Engineering, Arizona State University Tempe Arizona 85281 USA; b Institute of Electronics, Atomic Energy Research Establishment, Bangladesh Atomic Energy Commission Dhaka 1349 Bangladesh khalid.baec@gmail.com; c Department of Advanced Energy Engineering Science, Interdisciplinary Graduate School of Engineering Sciences, Kyushu University Fukuoka 816-8580 Japan khalid@kyudai.jp; d Department of Computer Science and Engineering, Daffodil International University Dhaka 1216 Bangladesh; e Department of Electrical and Electronic Engineering, Mymensingh Engineering College Mymensingh 2200 Bangladesh; f Department of Physics & Astronomy, East Texas A&M University Commerce TX 75428 USA; g Department of Mechanical Engineering, Faculty of Engineering and Technology, Jain (Deemed-to-be) University Bengaluru Karnataka-560069 India; h Department of Mechanical Engineering, Vivekananda Global University Jaipur Rajasthan 303012 India; i School of Engineering and Technology, Shobhit University Gangoh Uttar Pradesh 247341 India; j Department of Mechanical Engineering, Arka Jain University Jamshedpur Jharkhand 831001 India; k Centre for Research Impact & Outcome, Chitkara University Institute of Engineering and Technology, Chitkara University Rajpura Punjab 140401 India; l Department of Physics, College of Sciences, Taif University P. O. Box 11099 Taif 21944 Saudi Arabia; m Department of Chemistry, College of Science, University College of Taraba, Taif University Saudi Arabia; n Photonics and Photovoltaic Research Laboratory, Department of Physics and Material Science, Madan Mohan Malaviya University of Technology Gorakhpur 273010 U. P. India; o School of Chemical Engineering, Yeungnam University Gyeongsan 38541 Republic of Korea rajeshhaldhar.lpu@gmail.com

## Abstract

Emerging perovskite solar cells (PSCs) are facing environmental toxicity issues due to lead-based perovskites, and long-term stability remains a challenge. In recent years, silver bismuth iodides (Ag_3_Bi_1.1_I_6.3_) have gained attention as an absorber due to their lead-free, non-toxic, and cost-effective characteristics. However, device performance is still low so research is necessary to make it marketable. In this study, SCAPS-1D simulation is utilized to develop PSCs with an Ag_3_Bi_1.1_I_6.3_ absorber, C_6_TBTAPH_2_ Hole transport layer (HTL), and four distinct electron transport layers (ETLs) (STO, MZO, ZnSe, PC_60_BM) under standard illumination. In light of these factors, a thorough investigation of the FTO/ETL/Ag_3_Bi_1.1_I_6.3_/C_6_TBTAPH_2_/Au combination was conducted to evaluate the influence of power conversion efficiency (PCE). For each of the four assessed combinations, modifications were made to the absorber, the HTL, the ETL thickness, the acceptor density of the HTL, the acceptor doping, and the defect density of the absorber. The effects of these topologies on quantum efficiency, *J*–*V* characteristics, generation and recombination processes, series and shunt resistance, and temperature impact were also investigated. In the end, the most effective cell in this investigation was the FTO/MZO/Ag_3_Bi_1.1_I_6.3_/C_6_TBTAPH_2_/Au configuration with a PCE of 20.72%, *V*_OC_ of, *J*_SC_ of, and FF of at 300 K temperature. The previously described results have the potential to significantly advance the development of lead-free PSCs, which are more environmentally friendly and efficient, thereby opening the door for their eventual widespread use.

## Abstract 2

Here SCAPS-1D simulation is utilized to develop the PSCs, which comprise an Ag_3_Bi_1.1_I_6.3_ absorber, C_6_TBTAPH_2_ hole transport layer (HTL), and four distinct electron transport layers (ETLs) (STO, MZO, ZnSe, PC_60_BM) under standard illumination.

## Graphical abstract

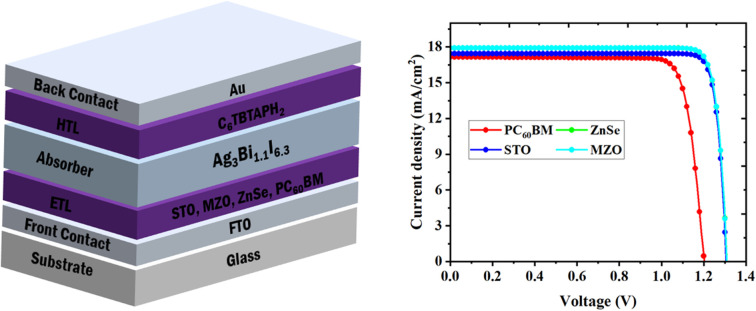

## 1 Introduction

Perovskite solar cells (PSCs) are considered one of the solutions to converting solar energy to usable electric energy. PSCs are widely used due to their laudable optoelectronic attributes, such as an admirable bandgap, an exalted absorption coefficient, high carrier mobility, a broad diffusion length, and an easy and cost-effective materials processing sequence.^1–3^ In addition, the power conversion efficiency (PCE) of PSCs, which has increased from 3.8% to 26.1% over the past decade, makes PSCs one of the most plausible options.^4,5^

However, despite having a high reported efficiency and cost-effective nature, PSCs still have not been commercialized due to toxicity and long-term stability issues.^6–10^ PSCs based on Pb specifically experience toxicity.^8,11^ Pb has consequences on the ecosystem and other life forms. For instance, even at low exposure limits, it causes serious harm to people, including functional abnormalities in the neurological, blood, kidney, and digestive systems, among others.^12–14^ When Pb-based perovskites are exposed to light, oxygen, moisture, or heat, they rapidly become unstable over time as a result of the polymorphism transition, water retention, or disintegration.^9,15^

Therefore, finding an alternative to Pb-free and efficient perovskite absorbers is a new challenge. As an alternative, scientists have employed various materials with identical electrical configurations to replace Pb. The most commonly used alternatives are tin (Sn^2+^) and germanium (Ge^2+^), as these materials belong to the same group and exhibit nearly identical characteristics.^16,17^ Nevertheless, Sn-based PSCs are readily oxidized from Sn^2+^ to Sn^4+^, and SnI_2_, one of the breakdown products, is just as dangerous as Pb.^18,19^ When exposed to air and moisture, Ge-based PSCs also exhibit a similar oxidation issue from Ge^2+^ to Ge^4+^.^19^ Therefore researchers have explored other materials in search of lower toxicity while aiming to maintain similar efficiency. Group VA elements such as antimony (Sb^3+^) and bismuth (Bi^3+^) have emerged as promising alternatives to lead-based materials due to their similar electronic properties. Both Bi^3+^ and Sb^3+^ possess the same ns^2^ electronic configuration as Pb^2+^ and exhibit greater stability under ambient conditions.^20^ However, while antimony is considered less toxic than lead, it is still not completely non-toxic, as highlighted by previous studies.^21,22^ The environmental and health risks associated with antimony must be thoroughly assessed, especially if these materials are to be utilized in large-scale applications. It is possible to create a bunch of Pb-free perovskite combinations by adding known organic and inorganic cations to the A-site, such as methylammonium (MA), formamidinium (FA), cesium (Cs), and Bi^3+^ or Sb^3+^ to the B-site, like MA_3_Bi_2_I_9_, FA_3_Bi_2_I_9_, and Cs_3_Bi_2_I_9_, but these materials often limit photon absorption at longer wavelengths due to their broader optical bandgap.^23^ Therefore, these materials are not sufficiently desirable to achieve high efficiency. However, substituting transition metals like silver (Ag^+^) for the A-site Ag_*a*_Bi_*b*_I_*a*+3*b*_, a silver bismuth iodide (SBI), provides an adjustable optical bandgap (1.65 to 1.85 eV), which is very advantageous for PSCs. The composition of SBI absorbers may be tuned by adjusting the AgI and BiI_3_ precursor ratio (changing the value of *a*, *b*), resulting in AgBiI_4_, Ag_2_BiI_5_, Ag_3_BiI_6_, and AgBi_2_I_7_.^24,25^

Recent studies have extensively explored bismuth-based lead-free perovskites for solar cell applications due to their ns^2^ lone pair configuration and environmental stability.^26,27^ Among these, compounds such as Cs_3_Bi_2_I_9_ and MA_3_Bi_2_I_9_ have received attention due to their non-toxicity and ease of processing. However, their large optical bandgaps (≈2.1–2.3 eV) significantly limit photon absorption in the visible region, resulting in low power conversion efficiencies (typically <1%).^23,28^ In contrast, Ag–Bi–I-based systems such as Ag_3_BiI_6_ offer a narrower, tunable bandgap (1.65–1.85 eV), superior film-forming quality, and stronger visible-light absorption—essential for efficient solar energy harvesting. Recent work by Simonov *et al.* and others has demonstrated that adjusting the AgI/BiI_3_ precursor ratio (*e.g.*, Ag_3_Bi_1.1_I_6.3_) can engineer improved optoelectronic properties, reduce defect densities, and enhance carrier lifetimes.^24,29,30^ Compared to Cs_3_Bi_2_I_9_, which often crystallizes in 0D or layered 2D motifs leading to localized charge carriers, the Ag–Bi–I systems favor 3D-like structures or extended frameworks that promote better charge transport and photovoltaic performance. These advantages make Ag_3_Bi_1.1_I_6.3_ a compelling candidate for next-generation lead-free perovskite solar cells.

Y. Kim *et al.* presented in 2016 the first solution-processed AgBi_2_I_7_ solar cells having device structure FTO/TiO_2_/AgBi_2_I_7_/P3HT/Au with a PCE of 1.22%, demonstrating remarkable resilience for over ten days beneath ambient conditions.^31^ In 2018, N. Pai *et al.* partially replaced a sulfide dianion in several SBI compositions (AgBiI_4_, Ag_2_BiI_5_, Ag_3_BiI_6_, and AgBi_2_I_7_), resulting in an improved PCE of 5.44% with Ag_3_BiI_6−2*x*_S_*x*_ (*x* = 4%).^24^ In conclusion, among other benefits, the Ag_3_BiI_6_ absorber delivers a higher PCE with outstanding long-term stability. To consider the benefits of SBI's inherent photostability and ensure the achievement of high efficiency in a single-cell structure and cutting-edge approaches, such as an excess ratio of bismuth (Bi/Ag = 1.1/3).^24,29^ However, several challenges exist in fabricating SbI-based perovskites, including controlling stoichiometry, achieving uniform film deposition, selecting suitable solvents, and optimizing post-processing techniques.^32–34^ The long-term instability of absorber material under light and heat exposure remains a significant issue, as noted in previous studies.^35,36^ This instability limits the practical fabrication of these materials in solar cells. The use of organic cations, such as methylammonium (MA), is another factor that compromises structural stability, particularly at elevated temperatures, where organic cation-based perovskites have been shown to be less stable than their inorganic counterparts. Thermogravimetric analysis (TGA) of a SBI material highlights its superior thermal stability when compared to organic cation-based systems.^37^ In contrast, Cs_3_Bi_3_I_9_, although more stable in ambient air than MA- and FA-based perovskites, faces a significant challenge due to its poor film-forming ability, which hampers its application in thin-film solar cell technology.^38^ When compared to these systems, AgBi_2_I_7_ films demonstrate superior stability, maintaining structural integrity and showing no signs of phase separation or degradation after 10 days of exposure to moisture and air.^39^ This suggests that AgBiI-based perovskites could offer better fabrication potential due to their enhanced stability, though improvements in stoichiometry control and film uniformity are still needed for scalability. PSCs comprised of an SBI of Ag_3_BiI_6_ with an excess ratio of bismuth (Bi/Ag = 1.1/3) exhibited a pinhole-free surface shape and enhanced carrier mobility by adjusting the stoichiometry of BiI_3_. The excellent electrical characteristics of PSCs with an absorber layer made of Ag_*a*_Bi_*b*_I_*a*+3*b*_ where, *a* = 3, *b* = 1.1 (Ag_3_Bi_1.1_I_6.3_) make them promising.^24,29^

In addition, selecting the right charge transportation layer can significantly enhance the device's efficiency and stability.^40–42^ Furthermore, solar metrics, including PCE, fill factor (FF), short-circuit current density (*J*_SC_), and open-circuit voltage (*V*_OC_), are extensively influenced by the thicknesses of the charge transport layer, as well as its interface and phase-matching properties.^43–46^ In particular, the Electron Transport Layer (ETL) is a crucial aspect of PSCs, as it removes electrons from the absorber and prevents holes from forming.^47,48^ The academic world has been closely monitoring the introduction of new elements through the previously mentioned pathway, particularly in ETL, to enhance the PCE of PSCs further. For PSCs, TiO_2_, ZnO, and SnO_2_ are meticulously investigated and in the research community, new ETLs remain a significant issues.^48–50^ Many ETL materials face these several issues such as photo-induced degradation and thermal instability, which can severely affect the longevity and operational stability of PSCs. Additionally, cost of ETL material is a critical concern in the selection of ETL materials for large-scale applications.^51,52^ Moreover, proper band alignment is crucial for efficient charge extraction, as it depends on factors such as the bandgap and electron affinity of the ETL material. The performance of a perovskite solar cell is heavily dependent on the proper selection of ETL materials, particularly in terms of band alignment between the absorber and the ETL. Poor alignment can lead to significant charge recombination, which lowers device performance.

PSCs without thermal treatment showed no photocurrent hysteresis when the as-deposited [6,6]-phenyl-C_60_-butyric acid methyl ester (PC_60_BM) layer was used.^53^ Because of its effective electron-accepting characteristics, which make electron extraction easier, PC_60_BM is often utilized as an ETL in PSCs. Its lowest unoccupied molecular orbital (LUMO) level is well-aligned with the conduction band of perovskites, ensuring smooth electron transmission. Furthermore, by passivating trap states, PC_60_BM lowers charge recombination between the solar cell's layers, increasing device efficiency. This passivation ability, along with its compatibility with various perovskite materials, makes PC_60_BM a solid alternative for boosting both the performance and stability of PSCs. Moreover, with gratitude to its strong electron mobility and 2.81 eV straight bandgap, zinc selenium (ZnSe) has been allocated as an ETL in PSCs and undoubtedly serves as an n-type collecting layer for trustworthy and fruitful commercial PSCs.^39^ Its excellent electronic properties, combined with the ability to facilitate efficient charge extraction, make ZnSe a potential material for improving both the efficiency and long-term stability of PSCs, paving the way for commercial applications. Researchers manufacture strontium titanate (STO) thin films through a combustion synthesis method, which produces excellent optoelectronic properties. By comparing the carrier-transport properties of STO to those of other electron-selective materials, they discover that STO is superior in electron extraction and also significantly extends the operational lifetime of devices. STO is an inorganic perovskite with an average direct electronic bandgap of 3.2 eV, which indicates that it absorbs considerably less UV radiation.^54,55^ Additionally, recent investigations on Mg-doped ZnO (MZO) and the consequences of Mg concentration on their opacity and structure have revealed that Mg-doped ZnO films are suitable for PSCs.^56^ Mg-doped ZnO (MZO) offers a unique advantage with its tunable bandgap energy. By controlling the magnesium concentration, the bandgap of MZO can be adjusted, which affects the energy levels of both the conduction band minimum (CBM) and the valence band maximum (VBM). An increase in magnesium concentration shifts the CBM higher and the VBM lower, improving electron selectivity. Furthermore, MZO thin films serve as efficient buffer layers, facilitating better charge carrier extraction, which contributes to higher device performance and stability.^57^

On the other hand, the hole transport layer (HTL) significantly impacts the efficacy, stability, and production costs of solar devices. Both organic and inorganic materials are used to choose the more economical, stable, and effective HTLs. Recent research, nevertheless, reveals that the greater band alignment, lower cost, and enhanced stability of inorganic and small molecule HTLs all contribute to better solar cell performance. In recent years, researchers have shown that octahexyltetrabenzotriaza porphyrin (C_6_TBTAPH_2_) is a very reliable HTL for PSCs.^58^ In the crystalline phases of non-peripherally substituted C_6_TBTAPH_2_, Q. D. Dao *et al.* observed comparatively strong hole drift mobility.^58^ Using the C_6_TBTAPH_2_ donors, organic solar cells have been successfully created using straightforward wet procedures. These desirable properties suggested that C_6_TBTAPH_2_ material would make an effective HTL for PSCs.^58^ Using the C_6_TBTAPH_2_ semiconductor as an HTL, we were able to show the PSC's manufacture, characterization, and modeling in this work.

In recent years, substantial progress has been made in understanding the optoelectronic behavior of lead-free perovskite materials through both experimental and computational studies. First-principles density functional theory (DFT) investigations have enabled detailed insight into the electronic structure, defect tolerance, and stability of various halide perovskites.^59,60^ Despite these advancements, Ag–Bi–I-based systems, such as Ag_3_Bi_1.1_I_6.3_, remain underexplored, especially from a simulation-driven solar cell optimization perspective, which motivates the present study.

In this article, we present the deposition and redaction of Ag_3_Bi_1.1_I_6.3_-based PSCs using an unparalleled device architecture: FTO/ETL/Ag_3_Bi_1.1_I_6.3_/C_6_TBTAPH_2_/Au. This analysis considers ZnSe, PC_60_BM, STO, and MZO as ETLs. The novelty of this work lies in the systematic SCAPS-1D-based simulation and optimization of Ag_3_Bi_1.1_I_6.3_-based perovskite solar cells, focusing on a wide range of electron and hole transport layers (ETLs/HTLs). While earlier reports have explored Bi-based perovskites, few have thoroughly evaluated the influence of functional layer selection, band alignment, and interface recombination effects in Ag–Bi–I systems. We provide in-depth analyses of the effects of doping degree and ETL/HTL layer thickness, absorber interface layers, electron/hole separation, absorber layer thickness, and absorber defect density on PV parameters using the SCAPS-1D in this work to maximize optimal cell output. In addition, the effects of operating temperature, *J*–*V*, QE, recombination rates, and series and shunt resistance were evaluated for PV performance generation. Lastly, the identified solar cell properties were used to compare with previous studies. The results reported here indicate that our approach to device optimization provides PSC researchers with a unique set of capabilities that can be applied to a relevant manufacturing technique in the lab, saving scientists both resources and time.

## 2 Device modeling and device structure

### 2.1 Device modeling

To analyze the device output characteristics mathematically, SCAPS-1D is one of the most reliable software.^61–65^ Due to its ability to solve the Poisson equations, SCAPS is frequently used in simulations of optoelectronic devices, particularly in the study of solar energy systems (eqn (1)) and the continuity equations (eqn (2) and (3)) may be used to estimate the output of PV devices. To model the device properties of PSCs, SCAPS-1D has been used in this study. A robust correlation between simulation and experimental data may be shown with SCAPS-1D. The Department of Electronics and Information Systems (ELIS) at the University of Gent created the optoelectronic device simulation program SCAPS-1D.^66^1
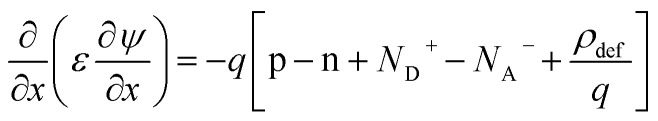
2
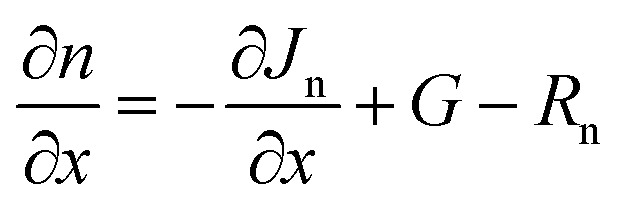
3
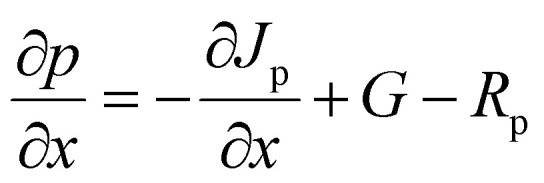
where *J*_n_ and *J*_p_ are considered to be electron and hole concentrations, respectively. Which are described in (eqn (4) and (5)).4
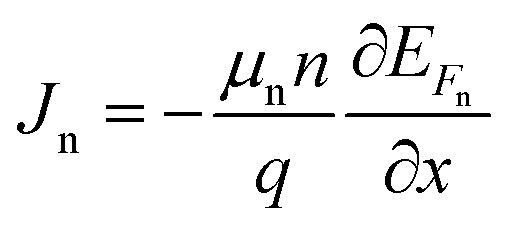
5
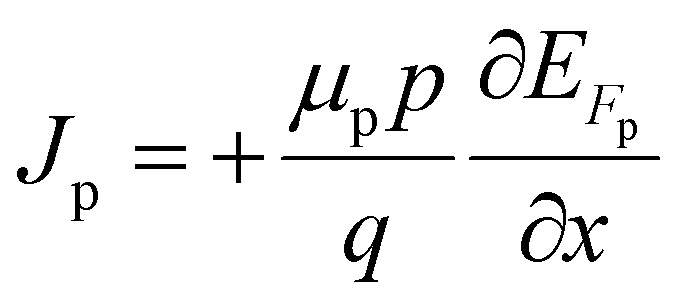


Seven different material layers as well as front and rear contact layers may be accepted by SCAPS-1D.^32^ In addition, the user-friendly options offered by SCAPS-1D, such as various defect energy distributions, complex defect shapes, and different defect charge types, provide a contextualized and ideal setting for this research. Using defect density and photovoltaic parameters (PCE, *J*_SC_, FF, and *V*_OC_), PSC properties may be predicted.^66^

### 2.2 Device structure

**Fig. 1:**
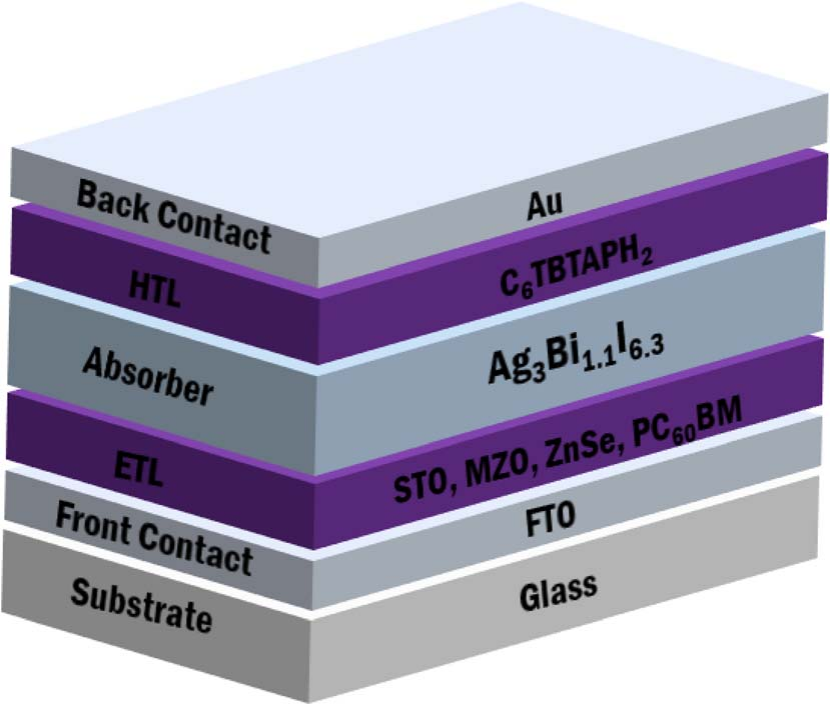
PSC structure based on Ag_3_Bi_1.1_I_6.3_ absorbers.

For this study, an n–i–p planar heterojunction structure including the gold (Au) back contact, transparent fluorine doped tin oxide (FTO), Ag_3_Bi_1.1_I_6.3_ absorbers, HTL, and ETL was simulated on SCAPS-1D, as Fig. 1 illustrates. The absorber layer Ag_3_Bi_1.1_I_6.3_ is located between the HTL and the ETL in every device construction. The HTL represents the p-region, the Ag_3_Bi_1.1_I_6.3_ absorbers the i-region, and the ETL the n-region. The absorber layer of the solar cell creates electron–hole pairs in response to light, with the electrons and holes traveling in the n- and p-layer respective directions. The electrical field that lies between the two layers allows electrons and holes to move and split.

The study investigates the impact and efficiency of four ETL-based optimized PSCs. Table 1 indicates the optoelectronic characteristics of the FTO, ETLs (STO, MZO, ZnSe, PC_60_BM), absorber layer (Ag_3_Bi_1.1_I_6.3_), and HTLs (C_6_TBTAPH_2_) as applied in the SCAPS-1D simulation in this research. The simulation operates at 300 K with radiation from a single sun (100 mW cm^−2^, AM 1.5G).

**Table 1:** Input parameters of the FTO, absorber, ETL, and HTL in this study

Material properties	FTO	Ag_3_Bi_1.1_I_6.3_	PC_60_BM	ZnSe	STO	MZO	C_6_TBTAPH_2_
Thickness (nm)	200	300	50	70	70	150	120
Bandgap, *E*_g_ (eV)	3.5	1.9	1.8	2.81	3.2	3.3	1.59
Electron affinity, *χ* (eV)	4.00	3.94	4.2	4.09	4	4	3.58
Relative dielectric permittivity, *ε*_r_	9.00	3.36	4	8.6	8.7	66	3
Conduction band effective density of states *N*_C_ (cm^−3^)	2.2 × 10^18^	2.2 × 10^19^	1 × 10^21^	2.2 × 10^18^	1.7 × 10^19^	2.2 × 10^18^	1.3 × 10^18^
Valence band effective density of states *N*_V_ (cm^−3^)	1.8 × 10^19^	2 × 10^19^	2 × 10^20^	1.8 × 10^18^	2 × 10^20^	1.8 × 10^19^	5.3 × 10^18^
Electron thermal velocity (cm s^−1^)	10^7^	10^7^	10^7^	10^7^	10^7^	10^7^	10^7^
Hole thermal velocity (cm s^−1^)	10^7^	10^7^	10^7^	10^7^	10^7^	10^7^	10^7^
Electron mobility, *μ*_n_ (cm^2^ V^−1^ s^−1^)	20	0.37	0.1	4 × 10^2^	5.3 × 10^3^	100	0.17
Hole mobility, *μ*_h_ (cm^2^ V^−1^ s^−1^)	10	85.31	0.1	1.1 × 10^2^	6.6 × 10^2^	25	0.17
Donor density, *N*_D_ (cm^−3^)	10^18^	0	1 × 10^17^	1 × 10^18^	2 × 10^16^	1 × 10^18^	0
Acceptor density, *N*_A_ (cm^−3^)	0	1 × 10^15^	0	0	0	0	2.2 × 10^18^
Total density (cm^−3^)	10^15^	3.36 × 10^14^	10^15^	1 × 10^15^	10^15^	10^15^	1 × 10^14^
References	67	29	68	69	67	69	58

## 3 Result and discussion

### 3.1 Band diagram

**Fig. 2:**
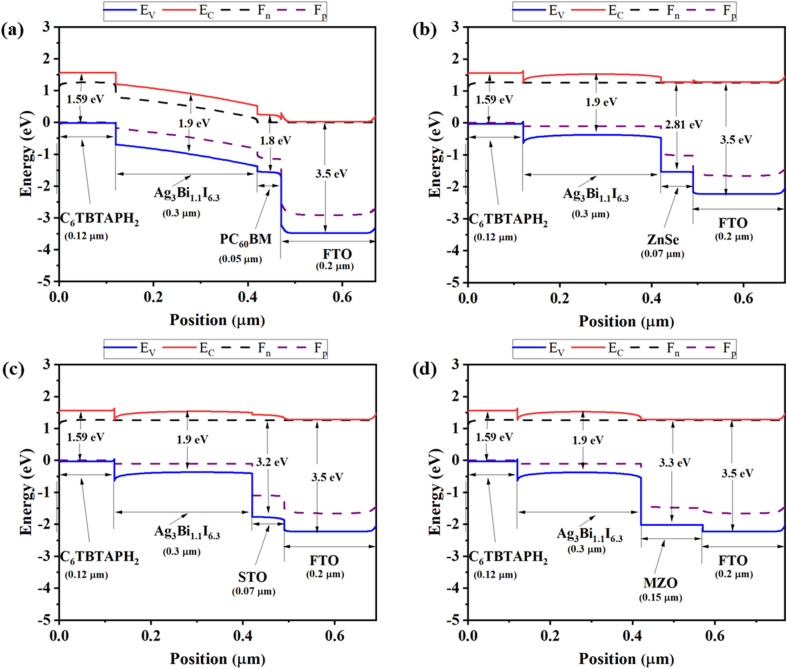
Energy band diagram of PSCs with distinct ETLs as (a) PC_60_BM, (b) ZnSe, (c) STO, (d) MZO.

In Fig. 2, band topologies with four ETLs are shown. The absorber layer and HTL are fixed; therefore, the only reason the band diagram varies is the ETL. Band alignment is necessary to enhance the device's functionality, as the extraction of electrons and holes directly impacts it.^70,71^ In most instances, the induced excitons produced by light irradiation go from the absorber layer to the ETL and HTL. The HTL's ionization energy should be lower than that of the absorber layer to quicken up the hole extraction process, while the ETL's electron affinity should be stronger than the absorber's to aid in the electron extraction process.^70^

When the device is not illuminated, it has only one Fermi level; however, when it is, it splits into two quasi-Fermi levels, one for the holes and one for the electrons. With these two quasi-fermi levels, coexisted the *E*_C_ and *E*_V_.^72^ As shown in Fig. 2, *E*_C_ and *F*_n_ both functioned in the same way. But *F*_P_ always lays above *E*_V_ for all ETLs. The Conduction Band Offset (CBO) and the Valence Band Offset (VBO), which can be either positive or negative, have significant consequences for PSCs.^73^ Owing to the decreased carrier recombination, the positive CBO (spike-like) where the ETL material's conduction band is higher than the absorber material has superior PCE.^74^ Because of the photo-generated carrier losses, efficiency and *J*_SC_ are reduced with a larger positive CBO. Conversely, when the ETL conduction band is lower than the active layer, negative band alignment, also known as Cliff-like band alignment, is seen. Every ETL forms a cliff with an absorber layer and exhibits comparable features.^75^ Comparably, when the valence band of the HTL material is higher than that of the absorber material, the HTL/active layer exhibits positive VBO (spike-like) and can increase *J*_SC_.^76^ Conversely, when the valence band of the HTL material is lower than that of the absorber material, the HTL/active layer exhibits negative VBO (cliff-like). The performance of this solar cell is directly impacted by the many Cliffs and spikes seen in Fig. 2.^76^

### 3.2 Optimization of device parameters

#### 3.2.1 Optimization of absorber thickness

**Fig. 3:**
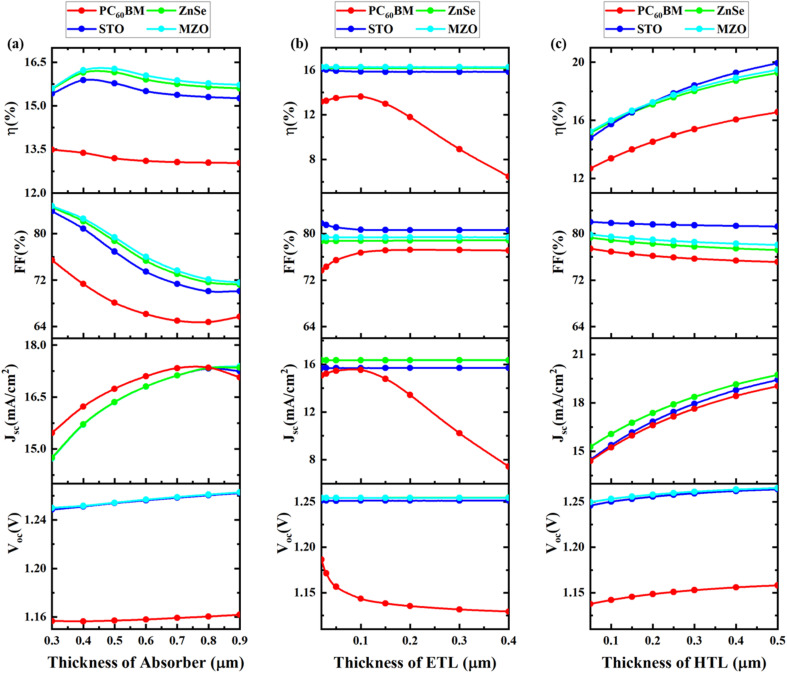
Impact of the (a) absorber, (b) ETL, and (c) HTL thickness on device performance.

The thickness of the absorption layer significantly affects PSC performance. Fig. 3 proposed PSCs architecture is analyzed by changing the absorber, ETL, and HTL thickness. It is significant to remember that different factors, including device architecture, and performance measures, might affect the ideal thickness.^77,78^ Adequate thickness selection is necessary to build the cell in any laboratory. Using FTO and Au as contact materials was the first step in the entire process. The absorber layer's thickness is varied between 0.3 μm and 0.9 μm to determine the optimal thickness, and the PV performance (*V*_OC_, *J*_SC_, FF, and PCE) is examined. The impact of altering the absorber layer's thickness on PV performance is seen in Fig. 3(a). When the thickness was raised, *V*_OC_ rose relatively little—from 1.15 V to 1.27 V—while *J*_SC_ increased—from 14.72 mA cm^−2^ to 17.38 mA cm^−2^—because it could absorb a significant percentage of the solar spectrum and produce a significant quantity of electron–hole pairs. However, FF is progressively reduced as absorber thickness increases, adding to the series resistance and leading to significant carrier recombination losses. At 0.5 μm, PCE rises to 16.27% from 13.49% and then begins to decline. The MZO ETL layer had the best efficiency while PC_60_BM had the lowest. High efficiency may be achieved by tuning as the absorber layer thickens. This results in improved cell efficiency because the photon-capturing capacity increases.^79^ However, as the thickness increases further, high-wavelength photons are absorbed, leading to quasi-neutral recombination and a decline in cell performance.^79^ The high resistance (series) of cells may be the cause of the rise in current density and fall in fill factor at increasing thicknesses. In line with earlier published research, 0.5 μm is believed to be the optimal absorber layer thickness.

#### 3.2.2 Optimization of ETL thickness

A sensitivity to photovoltaic performance was identified for the ETL thickness.^47^ When designing exceptionally efficient PSCs, ETL features should be carefully chosen. A good ETL can help increase transmittance and decrease recombination currents in PSCs. The fluctuation in performance characteristics at the thickness adjustment of ETL from 0.02 μm and 0.4 μm is shown in Fig. 3(b). It is noted that the PCE, FF, *J*_SC_, and *V*_OC_ are essentially constant. With PC_60_BM, the *V*_OC_ is 1.18 V, which is lower than with other ETLs, which are 1.25 V. When comparing *J*_SC_ with PC_60_BM to the other, it displays a lower. The FF with STO displays a greater value, but when comparing PCE with MZO and 0.1 μm thickness, it has a greater PCE (16.27%) than other ETLs. This is consistent with previously stated figures. This is in good agreement with previously stated figures.^47^

Among the evaluated ETLs, Mg-doped ZnO (MZO) demonstrated the highest PCE (16.27%) due to its favorable optoelectronic characteristics. MZO offers a tunable bandgap (3.2–3.7 eV) that depends on the Mg concentration, allowing for optimized conduction band alignment with the absorber layer. This tunability enhances electron selectivity and minimizes interfacial recombination.^80,81^ Furthermore, MZO is known for its low density of oxygen vacancies, which helps passivate interfacial defects, thereby improving charge extraction and device stability.^56,57^ In contrast, traditional ETLs such as TiO_2_ suffer from UV-induced photocatalytic degradation, slow electron mobility, and pronounced hysteresis effects, which degrade long-term device performance. SnO_2_, although exhibiting better mobility and lower temperature processing than TiO_2_, can present non-ideal energy level alignment and occasionally poor compatibility with certain absorber chemistries,^49^ MZO's combination of band tunability, high mobility, and low interfacial trap density makes it a strong candidate for next-generation ETLs in lead-free perovskite solar cells. Nevertheless, challenges such as ensuring uniform Mg incorporation and minimizing surface roughness during film deposition still require attention for experimental scalability.^82–84^

#### 3.2.3 Optimization of HTL thickness

The effect of HTL thickness on overall performance metrics is shown in Fig. 3(c), where ZnSe, PC_60_BM, STO, and MZO are used as ETLs, and the thickness of the C_6_TBTAPH2 HTL is varied. As the thickness of HTL grows from 0.05 μm to 0.5 μm, the *V*_OC_ rises from 1.13 V (PC_60_BM) to 1.26 V (MZO), as shown in Fig. 3(c). Additionally, the highest *J*_SC_ (16.3 mA cm^−2^) was achieved for MZO material. FF decreases with an increase in HTL thickness for all device structures. However, the PCE rose 19.94% (STO) and 19.48% (MZO) with an HTL thickness of 0.5 μm, indicating that this is the ideal HTL thickness for optimal performance. This is consistent with previously stated figures. This is consistent with established figures.^85,86^

While optimizing the thickness of the ETL and HTL layers, it is critical to recognize that the carrier diffusion length and built-in electric field strength across these layers fundamentally govern efficient charge extraction. In an ideal model, increasing the ETL or HTL thickness improves selectivity and reduces interfacial recombination. However, beyond a critical thickness, carrier extraction efficiency (CEE) begins to decrease due to enhanced series resistance and increased transit time for minority carriers. For instance, ETLs thicker than 100–150 nm can inhibit electron mobility, particularly in wide-bandgap materials like MZO, thereby degrading *J*_SC_ and FF. Similarly, overly thick HTLs may impair hole mobility and increase charge accumulation at the interface. Literature suggests that the optimal ETL/HTL thickness should remain below or comparable to the minority carrier diffusion length, allowing for a minimal potential barrier for carrier transfer at interfaces.^87–90^ Our thickness optimization (ranging from 0.02 to 0.4 μm for ETL and 0.05 to 0.5 μm from HTL) aligns with these theoretical efficiency limits, offering a balance between interfacial energy alignment and practical charge collection efficiency.

#### 3.2.4 Optimization of acceptor density of the absorber

**Fig. 4:**
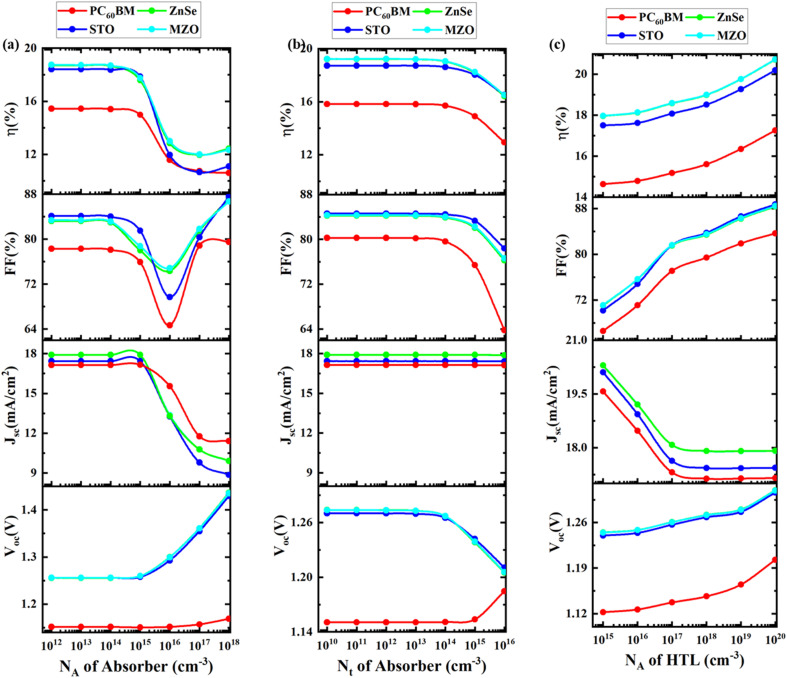
Impact of (a and b) absorber, and (c) HTL properties on PSC performance.

The absorber layer's acceptor density (*N*_A_) is responsible for capturing and transporting holes, which act as positive charge carriers. *N*_A_ in the absorber layer is a critical component influencing the photovoltaic cell's performance.^85,91^ Establishing an optimal acceptor density is necessary to balance the trade-offs of *V*_OC_, *J*_SC_, and FF. The impact of adjusting the absorber layer's *N*_A_ on PV parameters is seen in Fig. 4(a). While the *N*_A_ is changed between 10^12^ cm^−3^ and 10^18^ cm^−3^, the absorber thicknesses remain at 0.5 μm. *V*_OC_ levels are seen to rise in response to an increase in *N*_A_, but *J*_SC_ progressively falls. The absorber layer's *N*_A_ may affect the *V*_OC_. An increase in *N*_A_ will result in a greater *V*_OC_, as the *V*_OC_ and NA are directly related due to the acceptor states capturing holes and potentially creating a distinction between the absorber layers. The values of FF with MZO ETL are shown to increase between 83.37% and 86.7% as NA increases. At higher *N*_A_, it produces an electric field in the space charge zone, which lowers free carrier recombination. However, the PCE constant remains constant up to 10^14^ cm^−3^ and then progressively decreases from 17.89% to 9.9% as the *J*_SC_ starts to decrease. For our device, the ideal *N*_A_ is exactly 10^14^ cm^−3^, and this choice is consistent with the results documented in the literature.^79,85^

#### 3.2.5 Optimization of defect density of the absorber

The absorber layer's defect density (*N*_t_), which can act as recombination sites, can lower the number of free charge carriers and lower the PCE.^78,92,93^ It is a critical component that dictates how well the PSCs function. It is essential to decrease absorber layer defect density to lengthen carrier lifetime and minimize recombination losses. The impact of varying the *N*_t_ on PV characteristics is depicted in Fig. 4(b). While the *N*_A_ remains at 10^14^ cm^−3^, the *N*_t_ is adjusted from 10^10^ cm^−3^ to 10^16^ cm^−3^ to examine its impact on device performance. Up to 10^13^ cm^−3^, the characteristics seem to stay the same, but as *N*_t_ increases, they appear to decrease. However, despite constant *J*_SC_, *V*_OC_ decreases gradually as the absorber layer grows. When *N*_t_ reaches the value of 10^13^ cm^−3^, both FF and PCE fall precipitously, from 84.32% to 76.6% and 19.22% to 16.5%, respectively. As such, it makes sense to keep the *N*_t_ low. Based on the literature, the optimal *N*_t_ value was 10^14^ cm^−3^. In the absorber layer, the equivalent efficiencies reached were 19.22% and 19.05%, respectively, when defect concentrations of 10^13^ cm^−3^ and 10^14^ cm^−3^ were compared. Although the efficiencies appear comparable at both defect densities, selecting an optimal defect density of 10^14^ cm^−3^ is recommended, as it offers long-term stability, dependability, and the potential for future performance gains. The experimental results and theoretical arguments presented in the cited paper justify this value.^78,93^

#### 3.2.6 Impact of acceptor density of HTL

The acceptor concentration has a major impact on how the charge carriers produced by sunlight are separated.^44^ An electric field present at the absorber/HTL contacts, dependent on the acceptor density, sets these charge carriers apart.^85^ The change in *N*_A_ from 10^15^ cm^−3^ to 10^20^ cm^−3^ of C_6_TBTAPH_2_ as HTL, with all other optoelectronic parameters held constant, is shown in Fig. 4(c). With an increase in doping density, *J*_SC_ falls gradually, while *V*_OC_, FF, and PCE rise. The *V*_OC_ rises in tandem with the HTL's increasing *N*_A_. An increase in the intrinsic voltages at the HTL/perovskite interface, resulting in a rise in electric potential, is responsible for the higher *V*_OC_ value at higher *N*_A_. Strengthening charge carrier separation with less recombination raises the PSC's PCE. The optimal value for doping density is HTL 10^20^ cm^−3^ since it exhibits the best PCE. By putting this optimization in place, an additional assessment procedure is carried out.

### 3.3 Effects of various parameters on PV performance

#### 3.3.1 Effect of series resistance

**Fig. 5:**
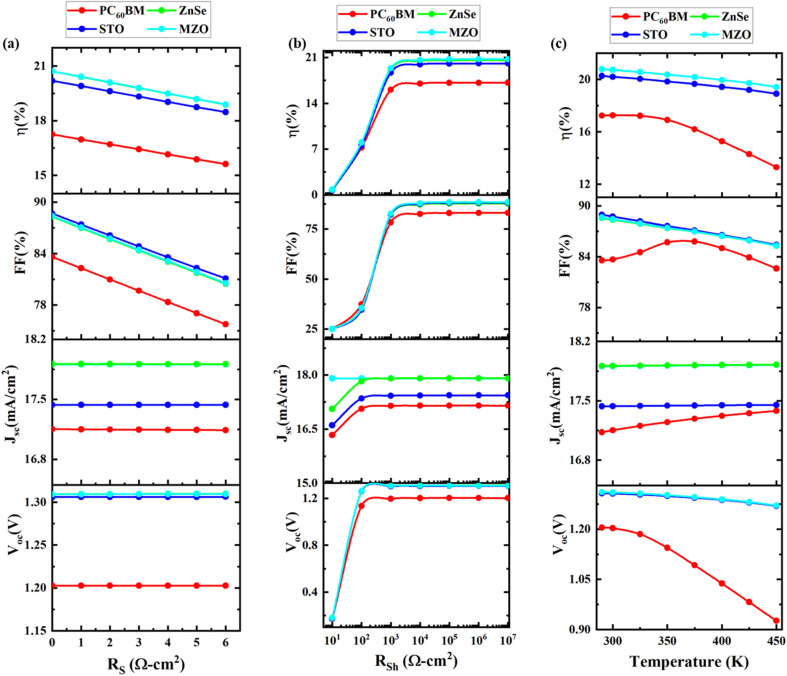
Impact of (a) series resistance, (b) shunt resistance, and (c) temperature on device performance.

Individual shunt (*R*_Sh_) and series (*R*_S_) resistances have a significant impact on the performance of the PSC, as they control the slopes and shapes of the current–voltage characteristics.^94,95^*R*_S_ is primarily caused by connections between the different layers of structures, metal contacts, the semiconductor–metal interface, and improper solar cell manufacturing procedures, which significantly impair perovskite performance.^96^ Moreover, solder bond degradation can significantly increase series resistance in PSCs. One of the primary contributors to *R*_S_ is the fluorine-doped tin oxide (FTO) layer where higher resistive losses occur resulting in the reduction of FF, thus limiting the overall performance of the PSCs. *R*_S_ reflects the electrical resistance encountered when connecting the device to external loads *via* its front and back contacts. The work function and thickness of the contact materials, as well as electron loss through scattering and recombination in the ETL, HTL, and perovskite layers, contribute to the overall *R*_S_ of the device.^97^ Minimizing *R*_S_ is crucial for maximizing PSC efficiency, as it directly affects charge extraction and overall device performance. To achieve this, various methods such as doping charge transport layers, applying interface modifiers, and optimizing fabrication techniques, have been employed to reduce *R*_S_, with proven results in improving PSC performance.^57,98–100^ In addition, redesigning the device geometry from square to rectangular and incorporating wrap-around tin busbars on the FTO electrode has been shown to shorten the charge transport distance, thus effectively reducing *R*_S_.^95,101^ In Fig. 5(a)*R*_S_ is changed from 0 Ω cm^2^ to 6 Ω cm^2^ to examine its influence on the PSC. *V*_OC_ and *J*_SC_ change very little in response to changes in *R*_S_, while FF drastically decreases, which causes PCE to fall for all four structures as *R*_S_ increases. Eqn (6) illustrates the JV properties of a heterojunction SC in a common diode model.^3^6
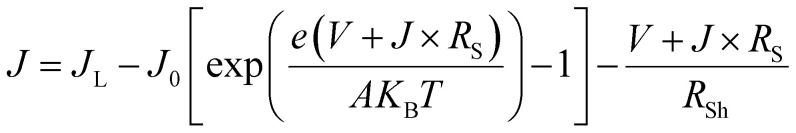
where *V* is the voltage, *J*_0_ is the reverse saturation current, *A* is the ideality factor, *J* = circuit current, *J*_L_ is the current caused by light absorption, and electron charge (*e*), the Boltzmann constant (*K*_B_), and temperature (*T*).

#### 3.3.2 Effect of shunt resistance

The resistance across the solar cell that allows current to bypass the active cell region and short-circuit the cell, thereby lowering its output power, is known as shunt resistance.^94,95^ The primary cause of shunt resistance in PSCs is recombination defects, often resulting from the formation of pinholes and cracks in the thin-film layers. These defects provide pathways for leakage current, which can significantly lower the PCE by diverting the photo-generated current away from the solar cell junction, thus reducing voltage.^73^ Surface passivation techniques, such as depositing ultra-thin layers at low temperatures, have been widely adopted to address these issues. Atomic layer deposition (ALD) has emerged as a popular method to provide high-quality, low-temperature passivation layers that not only enhance cell efficiency but also improve stability by protecting PSCs from environmental degradation.^74^ We change the value of *R*_SH_ from 10 Ω cm^2^ to 10^6^ Ω cm^2^ to investigate its impact on solar cell efficiency. Fig. 5(b) shows the performance characteristics that change as *R*_Sh_ increases. *R*_Sh_ is mostly caused by leakage current. PV characteristics gradually climb in tandem with an increase in shunt resistance. Consequently, a higher *R*_SH_ and a lower series resistance are required for increased cell efficiency. The fill factor, which assesses how well a solar cell converts sunlight into electrical power, might drop as a result of a low *R*_Sh_. This is due to the possibility of current leakage caused by the low *R*_Sh_, which might lower the cell's effective voltage and current output. It may raise the cell's dark current, which may lower the cell's power output and open-circuit voltage. This is because reduced shunt resistance may offer an alternative channel for current flow, thereby boosting current flow through the cell even when light is not present. Analogous patterns have been observed in earlier published works.^96,102^

#### 3.3.3 Effect of temperature

The operating temperature impacts the output parameters.^103,104^ The temperature of a solar cell can vary from 288 K to 320 K when exposed to the open air, however, it can reach higher temperatures in special cases. The effect of temperature variation on PV parameters is seen in Fig. 5(c). The temperature is adjusted between 300 K and 450 K to see the effect on the device's functionality. *V*_OC_ levels fall and *J*_SC_ levels rise with rising temperatures. Because there are more photons involved in the creation of electron–hole pairs at higher temperatures, *J*_SC_ increases as a material's band gap energy lowers. *V*_OC_ decreases as a result of rising saturation current density and increasing *J*_0_ with temperature. Eqn (7) illustrates the relationship between the open-circuit voltage and *J*_0_, *J*_SC_, and the energy band gap.^3^7
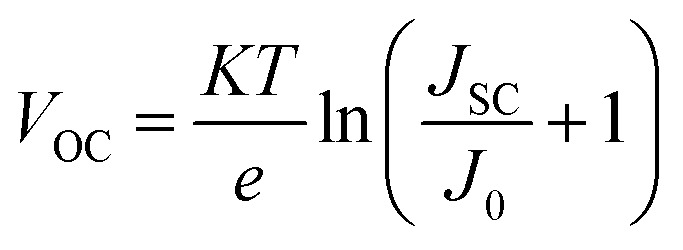
where electron charge (*e*) and Boltzmann constant (*K*). Additionally, it is seen that at increasing temperatures, the FF and cell performance decline. Furthermore, carrier mobility decreases as the temperature rises, increasing the cell's series resistance and reducing the fill factor. The decrease in *V*_OC_ and FF results in a decrease in the solar cell's overall efficiency. As a result, it may be concluded that room temperature is when solar cells operate most efficiently. Similar trends have been noted in previously published works.^104–107^

#### 3.3.4 Effect of absorber thickness with HTL thickness

**Fig. 6:**
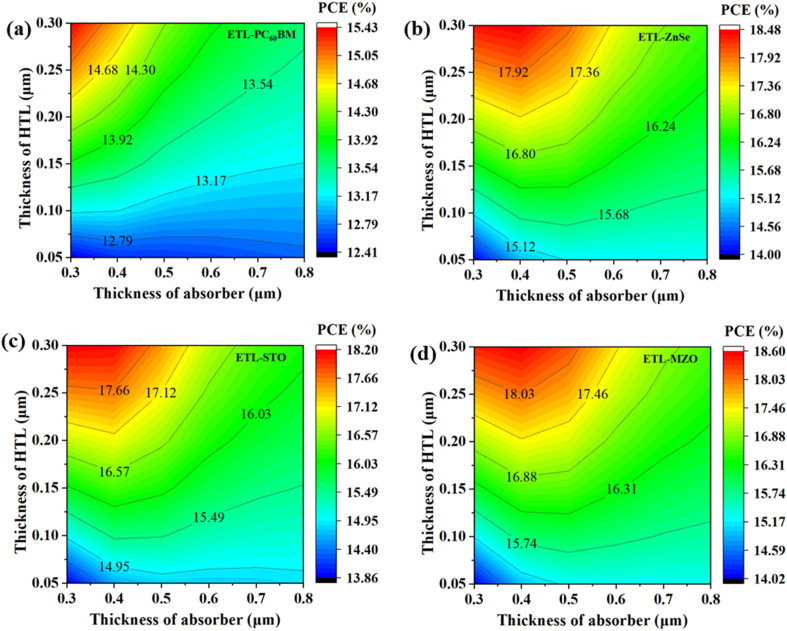
Relation between HTL and absorber thickness of (a) PC_60_BM, (b) ZnSe, (c) STO, and (d) MZO-ETL based device.

The fluctuation in PCE as a function of the HTL and absorber layer augmenting thickness is shown in Fig. 6. Regarding PCE, various HTLs display disparate phenomena. The PCE change for the structures of FTO/ETL/Ag_3_Bi_1.1_I_6.3_/C_6_TBTAPH_2_/Au is displayed in Fig. 6. The best PCE of 18.6% is provided by the devices FTO/MZO/Ag_3_Bi_1.1_I_6.3_/C_6_TBTAPH_2_/Au when the thickness of the absorber layer is maintained between 0.3 μm and 0.5 μm, with the HTL thicknesses of 0.25 μm and 0.3 μm, as shown in Fig. 6(d). For all ETL devices, the optimal thicknesses are observed in the upper-left corner of the figures, which correspond to lower absorber thickness values and relatively higher HTL thickness values. Fig. 6(d) depicts the structure of FTO/PC_60_BM/Ag_3_Bi_1.1_I_6.3_/C_6_TBTAPH_2_/Au, which offers the lowest PCE of 15.43% even when the absorber thickness ranges from 0.3 μm to 0.5 μm and the HTL thickness ranges from 0.25 μm to 0.3 μm. As the thickness of the absorber increases excessively, poor hole collection efficiency arises, leading to increased series resistance and enhanced recombination within the perovskite material. Conversely, a very thin perovskite layer limits photon absorption, resulting in a lower photocurrent.^108^ In contrast, while a thicker HTL can effectively cover an uneven perovskite surface, it may introduce additional challenges, such as increased series resistance.^85^ Balancing these thicknesses is critical for optimizing device performance and ensuring long-term stability.

#### 3.3.5 Effect of absorber acceptor density with absorber defect density

**Fig. 7:**
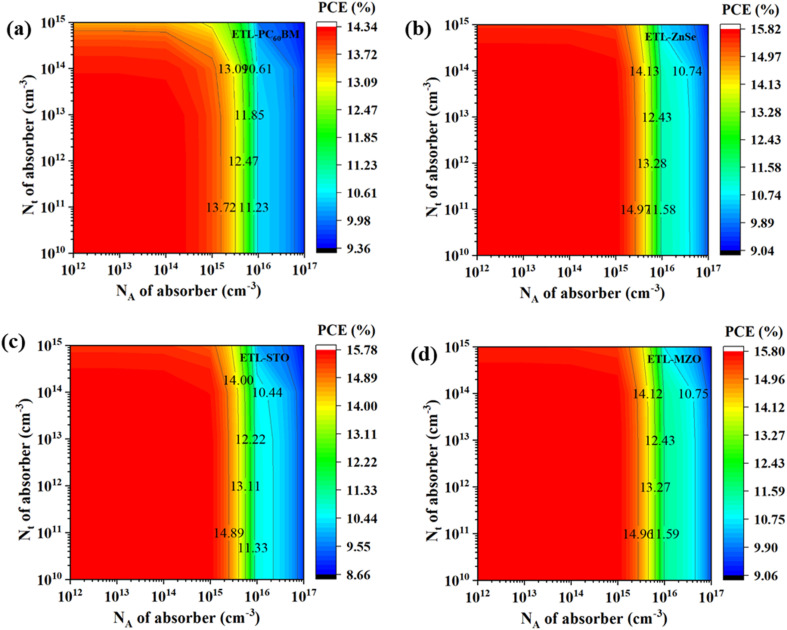
Relation between absorber acceptor density and defect density of (a) PC_60_BM, (b) ZnSe, (c) STO, and (d) MZO-ETL based device.

Fig. 7 illustrates how the fluctuations in absorber acceptor and defect density affect the PCE for the optimal combinations of absorber features. Acceptor density varies from 10^12^ cm^−3^ and 10^17^ cm^−3^, while defect density from 10^10^ cm^−3^ and 10^15^ cm^−3^. The best PCE of ∼15.8% is reported at an *N*_A_ of less than 10^12^ cm^−3^ for the structure of FTO/MZO/Ag_3_Bi_1.1_I_6.3_/C_6_TBTAPH_2_/Au, FTO/STO/Ag_3_Bi_1.1_I_6.3_/C_6_TBTAPH_2_/Au, FTO/ZnSe/Ag_3_Bi_1.1_I_6.3_/C_6_TBTAPH_2_/Au in Fig. 7(b)–(d), and it barely relies on the change in *N*_t_. The figure displays the lowest PCE of 14.34% for the FTO/PC_60_BM/Ag_3_Bi_1.1_I_6.3_/C_6_TBTAPH_2_/Au structure, where *N*_A_ and *N*_t_ are less than 10^15^ cm^−3^ and 10^14^ cm^−3^, respectively.

#### 3.3.6 JV and QE characteristics curve

**Fig. 8:**
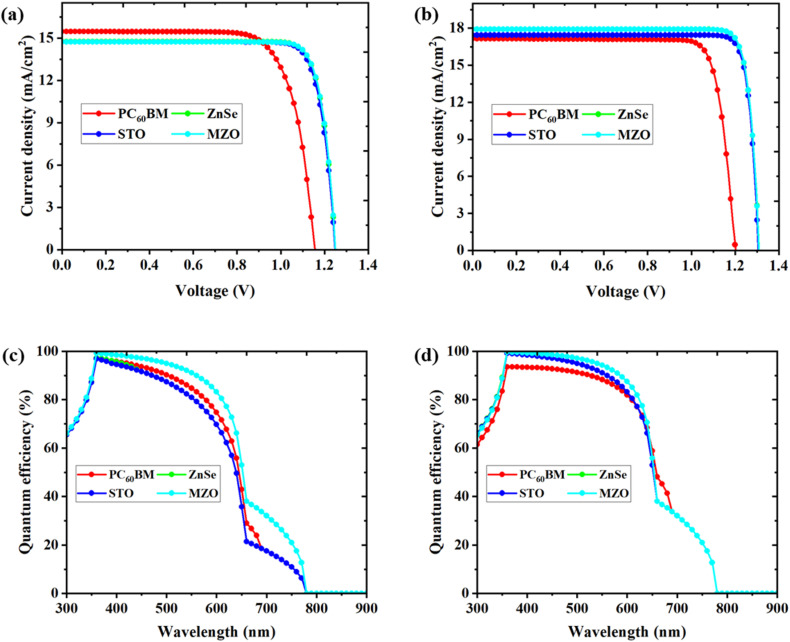
JV characteristics on device performance (a) before optimization and (b) after optimization, QE characteristics on device performance (c) before optimization and (d) after optimization.

JV and QE characteristics are obtained from Fig. 8 from four different structures. It is essential to note that this study has optimized several device parameters, including absorber thickness, acceptor density, defect density, ETL thickness, and HTL thickness, to enhance device performance. Prior to optimization, the *J*–*V* curves in Fig. 8(a) and (b) for all four ETLs display nearly the same value. For every ETL under study, the same photo-generated current in the device causes the same current density. The band structure of ETLs is frequently the cause of the variance. The MZO-based device outperforms the others in terms of *V*_OC_ and *J*_SC_. After device modification, a notable improvement is observed in the STO ETL device, which achieves an impressive *V*_OC_, albeit still comparatively lower than that of the MZO device.

A device's quantum efficiency (QE) measures how many photons are converted into current when illuminated with various light wavelengths. Two types of quantum efficiency are commonly used to evaluate a device's photocurrent generation capacity: internal and external QE. Internal quantum efficiency is the ratio of electron–hole pairs created to photons absorbed in a device's active layer. This may be used to determine the amount of photocurrent generated by photon absorption. Fig. 8(c) and (d) displays the QE curves for the first and final devices and illustrates the relationship between wavelength and QE. The finished device has been demonstrated to perform exceptionally well in the 350 nm to 550 nm wavelength range.

#### 3.3.7 Generation and recombination rates

**Fig. 9:**
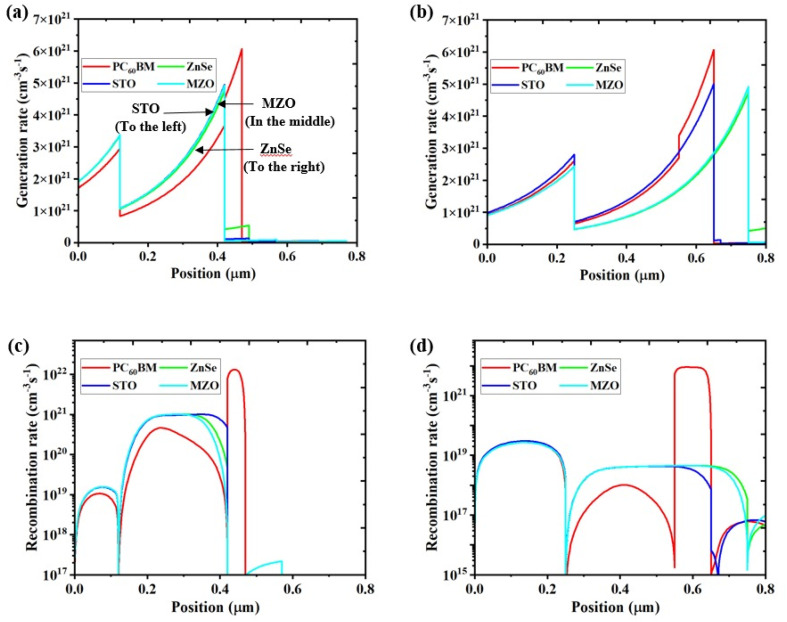
(a and b) Generation rate of the device before and after optimization, (c and d) Recombination rate of the device before and after optimization.

Fig. 9 illustrates how the cell's location within the apparatus affects the rate at which charge carriers are generated and recombined, thereby influencing cell performance. Excitons, or electron–hole pairs, are produced when electrons move from the valence band to the conduction band in a solar cell that is illuminated.^96^ More light absorption from a larger active layer results in the production of more charge carriers. The maximum rate of electron creation occurs in the region where most photons are absorbed, resulting in the device generating a substantial amount of charge. However, the shorter diffusion length of these charge carriers, which restricts their capacity to reach the corresponding electrodes efficiently, increases the chance of recombination in thicker layers. SCAPS-1D calculates the production of charge carriers based on the incident photon flux. *N*_phot_ (*λ*,*x*), which is specified in eqn (8), is used to determine the creation of the electron–hole pair *G*(*x*).^3^

8 *G*(*λ*,*x*) = *α*(*λ*,*x*)·*N*_phot_(*λ*,*x*)

The creation of charge carriers for initial and optimized devices is depicted in Fig. 9(a) and (b), respectively, at various positions within the range of 0 μm to 0.8 μm. PC_60_BM has the greatest generation rates at locations of 0.48 μm and 0.65 μm before and after device tuning, whereas MZO shows the lowest generation.

Carrier recombination, the reverse of carrier creation, is the process by which electrons from the conduction band go to the valence band and recombine with holes to become stable. Three primary mechanisms are involved in recombination: auger recombination, radiative recombination, and non-radiative recombination *via* defect states.^109^ Non-radiative and interface recombination processes strongly influence *V*_OC_ and FF in solar cells. Defect-related recombination within the bulk of the perovskite and at the interfaces can significantly degrade not only the PCE but also lead to hysteresis and long-term stability issues in PSCs. While it is widely accepted that non-radiative and interface recombination are major contributors to losses in *V*_OC_ and FF, there remains a lack of comprehensive and systematic understanding of the precise origins of recombination mechanisms in perovskite solar cells. A device's performance is affected by carrier recombination in the absorber layer and a shorter carrier lifetime when the defect rate increases. The devices with MZO ETL exhibit a notable decrease in recombination during device parameter optimization, whereas PC_60_BM remains essentially unchanged from its initial state, as depicted in Fig. 9(c) and (d). The electron–hole recombination rate throughout the device is affected by energy levels, and the distribution of recombination rates can be caused by flaws and grain boundaries.^110^

### 3.4 Final optimized device and comparison with previous study

By varying the composition ratios, various absorbers were designed, including Ag_2_Bi_3_I_11_, AgBi_2_I_7_, AgBiI_4_, Ag_2_BiI_5_, Ag_3_BiI_6_, and AgBi_2_I_7_. Experimental work has been conducted using Ag_3_BiI_6_ as the absorber, where the maximum PCE achieved was 4.30%, using TiO_2_ as the ETL and PTTA as the HTL.^111^ However, limited theoretical work has been done on this absorber. One numerical study reported a maximum PCE of 17.77% using an Ag_3_Bi_1.1_I_6.3_ composition as the absorber with CeO_*X*_ as the ETL.^29^ Our theoretical study, however, surpasses all previous theoretical records for the SBI (Ag_3_Bi_1.1_I_6.3_) absorber, achieving a PCE of 20.73% using MZO as the ETL and C_6_TBTAPH_2_ as the HTL. The superior performance of our solar cell devices is attributed to proper band alignment with the absorber, and the choice of materials for the ETL and HTL, which are more effective than those used in previous studies. Notably, all four devices investigated in this study are theoretically superior in performance compared to previously reported SBI absorber-based devices, as shown by the comparison of PV parameters in Table 2. It is evident from Table 2 that a disparity exists between the experimental and simulation results, and several factors must be considered to explain this discrepancy. SCAPS-1D simulations sometimes assume idealized device structures, which significantly differ from the material non-uniformities, grain boundaries, and defects commonly present in experimental devices.^112–114^ These imperfections contribute to poorer performance by promoting charge carrier recombination and limiting mobility. Devices with smaller grain sizes, in particular, often exhibit reduced PCE due to increased grain boundary recombination.^114,115^ Additionally, the synthesized material's band gap may not match the theoretical or previously reported experimental values,^116,117^ which is another key reason for the observed disparity between PV performance in experimental and simulated results.

**Table 2:** Comparison of PV parameters of previous work with this work^a^

Type	Device structure	*J*_SC_ (mA cm^−2^)	*V*_OC_ (V)	FF (%)	PCE (%)	Ref.
E	FTO/TiO_2_/Ag_3_BiI_6_/PTAA/Ag	5.35	0.71	65.3	2.60	118
E	FTO/TiO_2_/Ag_3_BiI_6_/PTAA/Au	10.70	0.63	64.0	4.30	111
E	FTO/TiO_2_/Ag_3_BiI_6_/P3HT/Au	5.50	0.60	70.0	2.32	119
E	FTO/TiO_2_/Ag_3_BiI_6_/PTAA/Au	2.36	0.65	70.0	1.08	120
E	FTO/TiO_2_/AgBi_0.5_Sb_1.5_I_7_/PTB7/MoO_3_/Au	5.66	0.53	59.0	1.76	121
E	ITO/SnO_2_/AgBiI_4_/PTAA/Au	5.07	0.83	66.5	2.80	122
E	FTO/TiO_2_/AgBi_2_I_7_/P3HT/Au	3.30	0.56	67.4	1.22	39
E	FTO/TiO_2_/Ag_2_BiI_5_/P3HT/Au	6.80	0.49	63.0	2.10	123
E	FTO/TiO_2_/Ag_2_BiI_5_/PTAA/Au	6.04	0.69	62.4	2.60	34
T	FTO/CeO_*x*_/Ag_3_BiI_6_/Cu_2_O/Au	15.38	1.28	80.1	15.98	29
T	FTO/CeO_*x*_/Ag_3_BiI_6_/Te–Cu_2_O/Au	15.39	1.33	81.3	16.66	29
T	FTO/CeO_*x*_/Ag_3_BiI_6_/Se/Te–Cu_2_O/Au	15.41	1.39	82.6	17.77	29
T	FTO/PCBM/Ag_3_Bi_1.1_I_6.3_/C_6_TBTAPH_2_/Au	17.15153	1.2027	83.66	17.26	*
T	FTO/ZnSe/Ag_3_Bi_1.1_I_6.3_/C_6_TBTAPH_2_/Au	17.91245	1.3093	88.34	20.72	*
T	FTO/STO/Ag_3_Bi_1.1_I_6.3_/C_6_TBTAPH_2_/Au	17.4354	1.306	88.73	20.2	*
T	FTO/MZO/Ag_3_Bi_1.1_I_6.3_/C_6_TBTAPH_2_/Au	17.90747	1.3091	88.42	20.73	*

a T = theoretical; E = experimental; * = this work.

It is also important to note that real-world experimental results often deviate from theoretical predictions due to a range of non-ideal factors. Prior studies have shown that factors like rheology-driven thickness variations and bandgap shifts in electron transport layers (ETLs) can substantially influence photovoltaic performance, causing simulation-experiment mismatches even under identical device architectures.^124^ These insights emphasize the importance of correlating simulation results with carefully controlled experimental calibrations, which we aim to pursue in our future work.

## 4 Conclusions and future outline

Using the SCAPS modeling approach, this work explains the optoelectronic and photovoltaic characteristics of Ag_3_Bi_1.1_I_6.3_ perovskites. In conclusion, we examine how variations in the absorber, HTL, and ETL thickness impact the PV characteristics of the top four designs. Notably, PCE increases as an absorber, and HTL thicknesses rise, whereas ETL thickness has no effect. In addition, we study the defect density and absorber acceptor density to assess the effect on the devices' performance. The architecture FTO/MZO/Ag_3_Bi_1.1_I_6.3_/C_6_TBTAPH_2_/Au is the most efficient with a 20.72% efficiency, while the structure FTO/PC_60_BM/Ag_3_Bi_1.1_I_6.3_/C_6_TBTAPH_2_/Au has the lowest efficiency of the four at 17.26%. The study examines the impact of temperature, shunt resistance, and series resistance on PSCs, including their rates of recombination and production. The performance of all four PSCs is further validated by JV and QE characteristics. These results can potentially lead to the development of lead-free PSCs that are more economically feasible and efficient, opening the door for their incorporation into other applications. Additionally, by investigating material and interface engineering, stability enhancement, device design optimization, and utilizing sophisticated characterization techniques to improve Ag_3_Bi_1.1_I_6.3_ PSC performance.

Finally, this study is a preliminary theoretical investigation based on SCAPS-1D simulations. While the predicted device parameters show promising efficiency, they represent an idealized model and do not account for fabrication-dependent factors such as surface roughness, trap-assisted recombination, or degradation mechanisms. As such, experimental validation including *J*–*V* curve analysis, steady-state power output, and operational stability testing remains essential. Future work will focus on synthesizing Ag_3_Bi_1.1_I_6.3_-based perovskite solar cells with the optimal ETL and HTL configurations proposed here, enabling detailed comparison with the simulated performance and providing insights into real-world device limitations.

## Author contributions

M. K. Hossain, and: conceptualization, methodology, software, validation, formal analysis, investigation, data curation, writing – original draft, writing – review & editing, supervision, project administration; M. A. Islam, M. S. Uddin, A. K. Datta, and S. Islam: software, validation, formal analysis, investigation, data curation, writing – original draft; P. S. Bains, R. Sharma, A. Rajiv, A. M. S. Alhuthali, M. H. Abdellattif, D. K. Dwivedi, and R. Haldhar: validation, formal analysis, writing – review & editing.

## Conflicts of interest

The authors declare that they have no known competing financial interests or personal relationships that could have appeared to influence the work reported in this paper.

## Funding

The research was funded by Taif University Saudi Arabia project number TU-DSPP-2024-19.

## Data Availability

The raw/processed data required to reproduce these findings cannot be shared at this time as the data also forms part of an ongoing study and are available from the corresponding author on reasonable request.
